# Le dispositif intra-utérin: à propos d’une complication rare et revue de la littérature

**DOI:** 10.11604/pamj.2017.27.193.13106

**Published:** 2017-07-13

**Authors:** Adil Kallat, Ahmed Ibrahimi, Otheman Fahsi, Hachem El Sayegh, Ali Iken, Lounis Benslimane, Yassine Nouini

**Affiliations:** 1 Service d’'Urologie A, Hôpital Ibn Sina, CHU, Rabat, Maroc

**Keywords:** Migration, dispositif intra-utérin, vessie, Migration, intrauterine device, bladder

## Abstract

Le dispositif intra-utérin (DIU) constitue la méthode contraceptive la plus utilisée dans le monde. Sa migration trans-utérine est une complication rare et son incidence est estimée dans la littérature de 1/350 insertions à 1/10000 insertions. Nous rapportons le cas d'une patiente âgée de 40 ans, chez qui un DIU a été posé il y a 12 ans environ, ayant consulté pour des douleurs pelviennes et des lombalgies droites associées à une hématurie intermittente et des brûlures mictionnelles. Le bilan radiologique a mis en évidence un dispositif intra utérin calcifié en intra vésicale. Une cystotithotomie a été réalisée sans difficulté particulière permettant l'extraction du calcul et du DIU. Une sonde urinaire a été laissée en place pendant 5 jours puis retirée. Les suites opératoires ont été simples.

## Abstract (en)

The intrauterine device (IUD) is the most common contraceptive method used in the world. Transuterine migration is a rare complication, accounting for 1/350 - 1/10000 insertions in the literature. We report the case of a 40-year old patient, who had had an IUD insertion 12-year before, presenting with pelvic and right lower back pain associated with intermittent hematuria and burning during urination. Radiological assessment showed calcific deposits on intra bladder IUD. The patient underwent cystostomy, without any difficulty, allowing stone and IUD extraction. A urinary catheter was left in place for 5 days and then withdrawn. The postoperative course was uneventful.

## Introduction

Le dispositif intra-utérin (DIU) constitue la méthode contraceptive la plus utilisée dans le monde. Sa migration trans-utérine est une complication rare et son incidence est estimée dans la littérature de 1/350 insertions à 1/10000 insertions. Elle se fait le plus souvent vers la cavité abdominale. Plus rarement, elle se fait vers le pelvis. Dans ce cas la vessie est la plus fréquemment atteinte. La complication la plus fréquente est la formation d'une calcul.

## Patient et observation

**Figure 1:**
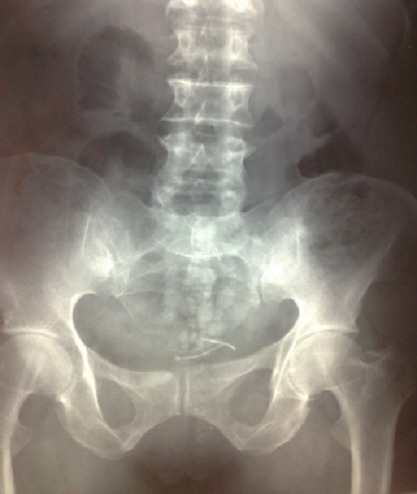
AUSP (arbre urinaire sans préparation) montrant une image de tonalité calcique associée à une formation en T au niveau de l’aire pelvienne évoquant un DIU

**Figure 2:**
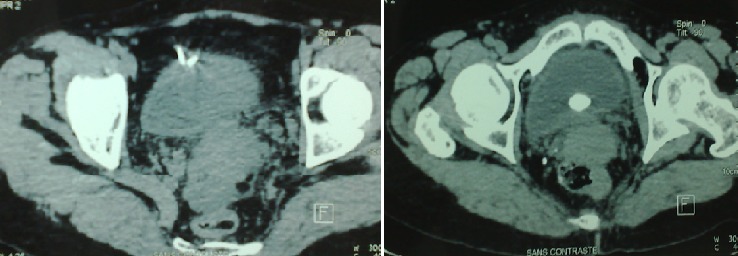
TDM (tomodensitométrie) abdomino-pelvienne montrant le DIU en intra vésical associé à un calcul

**Figure 3:**
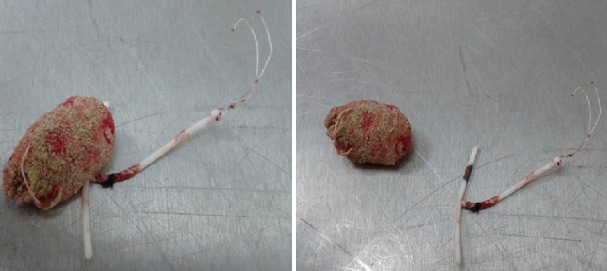
Aspect du DIU associé au calcul vésical après son extraction par voie chirurgicale

Nous rapportons le cas d'une patiente âgée de 40 ans, sans antécédents pathologiques particuliers, chez qui un DIU a été posé il y a 12 ans environ, ayant consulté pour des douleurs pelviennes et des lombalgies droites associées à une hématurie intermittente et des brûlures mictionnelles. Le début de la symptomatologie remonte à 1 an par l'installation de douleurs pelviennes et des lombalgies droites associées à une hématurie intermittente et des brûlures mictionnelles. Le bilan biologique était sans particularité avec un ECBU (examen cytobactériologique des urines) stérile et une leucocyturie sans bactériurie. La fonction rénale était normale. L'AUSP (arbre urinaire sans préparation) (Figure 1) a objectivé sur l'aire pelvienne une opacité de tonalité calcique latéralisée à droite et un DIU. L'échographie vésico-rénale a révélé une dilatation modérée des cavités pyélocalicielles du côté droit avec un calcul intravésical de 10mm environ. La tomodensitométrie abdomino-pelvienne (Figure 2) a montré une urétéro-hydronéphrose du côté droit avec un calcul intravésical et le DIU était en extra-utérin. Il a été donc décidé de réaliser l'ablation du DIU par voie chirurgicale. Une cystotithotomie par voie ouverte a été réalisée sans difficulté particulière avec extraction du calcul et du DIU (Figure 3). Une sonde urinaire a été laissée en place pendant 5 jours puis retirée. Les suites opératoires ont été simples.

## Discussion

La perforation partielle ou totale du myomètre lors de la pose du DIU est d'autant plus fréquente qu'il existe une anomalie du myomètre (utérus cicatriciel en particulier) ou en raison d'une utérus hypoplasique, d'une rétroversion ou d'une hyper anté-version méconnue [1]. Une migration secondaire sera favorisée par l'inflammation locale entraînée par les DIU au cuivre [2]. La patiente peut être asymptomatique. Dans le cas contraire, elle peut présenter des symptômes d'irritation urinaire à répétition, voire une hématurie terminale ou des douleurs pelviennes [3]. En cas de fistule vésico-utérine, la patiente peut signaler des menstruations vésicales, des urinations vaginales, une aménorrhée et/ou une incontinence urinaire [4]. L'ASP met en évidence un DIU mal positionné en dehors de l'aire de projection de l'utérus [5]. L'échographie pelvienne aide à la localisation de DIU [5, 6]. La cystoscopie permet de rechercher la présence de calcul et de déterminer si la migration intra vésicale est partielle ou totale. L'épreuve au bleu de méthylène ou une cystographie permettent de vérifier l'absence de fistule uro-génitale [6]. Dans la majorité des cas, l'ablation du matériel se fait sans difficulté lors de la cystoscopie en cas de localisation totalement intra-vésicale [3]. Ce geste pourra être précédé si nécessaire d'une lithotritie extra corporelle ou in situ en cas de calcul associé [6].

## Conclusion

La migration trans-utéro-vésicale d'une DIU peut être révélée par une symptomatologie clinique traduisant sa complication. Une prise en charge adaptée nécessite de bien localiser le DIU et de rechercher les éventuelles complications associées.

## Conflits d’intérêts

Les auteurs ne déclarent aucun conflit d'intérêt.
